# Applying the Tailored Implementation in Chronic Diseases framework to inform implementation of the Preferences Elicited and Respected for Seriously Ill Veterans through enhanced decision-making program in the United States Veterans Health Administration

**DOI:** 10.3389/frhs.2022.935341

**Published:** 2022-09-02

**Authors:** Leah M. Haverhals, Kate H. Magid, Jennifer Kononowech

**Affiliations:** ^1^Denver-Seattle VA Center of Innovation for Value Driven and Veteran-Centric Care, Rocky Mountain Regional VA Medical Center at VA Eastern Colorado Health Care System, Aurora, CO, United States; ^2^Health Care Policy and Research, School of Medicine, University of Colorado Anschutz Medical Campus, Aurora, CO, United States; ^3^VA Ann Arbor Health Care System, Ann Arbor, MI, United States

**Keywords:** life-sustaining treatments, Veterans, goals of care conversations, tailoring, implementation science

## Abstract

In 2017, the National Center for Ethics in Health Care for the United States Department of Veterans Affairs (VA) commenced national roll-out of the Life-Sustaining Treatment Decisions Initiative. This national VA initiative aimed to promote personalized, proactive, patient-driven care for seriously ill Veterans by documenting Veterans' goals and preferences for life-sustaining treatments in a durable electronic health record note template known as the life-sustaining treatment template. The Preferences Elicited and Respected for Seriously Ill Veterans through Enhanced Decision-Making (PERSIVED) quality improvement program was created to address the high variation in life-sustaining treatment template completion in VA Home Based Primary Care (HBPC) and Community Nursing Home programs. This manuscript describes the program that focuses on improving life sustaining treatment template completion rates amongst HBPC programs. To increase life-sustaining treatment template completion for Veterans receiving care from HBPC programs, the PERSIVED team applies two implementation strategies: audit with feedback and implementation facilitation. The PERSIVED team conducts semi-structured interviews, needs assessments, and process mapping with HBPC programs in order to identify barriers and facilitators to life-sustaining treatment template completion to inform tailored facilitation. Our interview data is analyzed using the Tailored Implementation in Chronic Diseases (TICD) framework, which identifies 57 determinants that might influence practice or implementation of interventions. To quickly synthesize and use baseline data to inform the tailored implementation plan, we adapted a rapid analysis process for our purposes. This paper describes a six-step process for conducting and analyzing baseline interviews through applying the TICD that can be applied and adapted by implementation scientists to rapidly inform tailoring of implementation facilitation.

## Introduction

In 2017, the United States Department of Veterans Affairs (VA) National Center for Ethics in Health Care began implementing the Life-Sustaining Treatment Decisions Initiative (LSTDI) (1). The purpose of the LSTDI is to encourage VA clinicians across the national, integrated health care system to conduct goals of care conversations (2) with seriously ill Veterans and/or their caregivers to identify Veterans' preferences for life-sustaining treatments (3). In turn, these efforts ensure that Veterans' goals and preferences are documented in the electronic health record in a standardized, durable note template called the life-sustaining treatment template (4). Documenting Veterans' preferences in the life-sustaining treatment template ensures the order set corresponding to the note is easily accessible across the entire VA healthcare system. According to the VA National Center for Ethics Life-Sustaining Treatment Report, as of March 2022, there were close to 800,000 total goals of care conversations documented in life-sustaining treatment template and order sets (5). While prior analyses conducted since the implementation of the LSTDI found that older Veterans and those at highest risk for hospitalization or death were most likely to have a documented life-sustaining treatment template, there remains room to improve documentation for this population (3).

In late 2020, the Preferences Elicited and Respected for Seriously Ill Veterans through Enhanced Decision-Making (PERSIVED) VA Quality Enhancement Research Initiative or QUERI, quality improvement program was developed to address high variation in completion of life-sustaining treatment templates for seriously ill Veterans. The PERSIVED QUERI was established following the completion of the VA Long-Term Care QUERI program (3, 6, 7). The Long-Term Care QUERI supported implementation of the LSTDI in VA's long-term care settings, which included VA nursing homes, known as Community Living Centers, and VA Home Based Primary Care (HBPC) programs (2). The PERSIVED QUERI is a five-year long quality improvement program which includes two projects; one that targets Veterans receiving VA HBPC, and the other that targets Veterans in contracted community nursing homes (8). In this manuscript, we focus on the HBPC arm of PERSIVED, specifically outlining how we are applying the Tailored Implementation in Chronic Diseases (TICD) framework to analyze pre-implementation interviews and inform the tailoring of implementation facilitation efforts to each HBPC program participating in PERSIVED. This approach can then be applied and tailored by implementation scientists, evaluators, and researchers to rapidly inform future health services interventions or initiatives.

## PERSIVED project context

### HBPC background

HBPC programs exist across all 171 VA Medical Centers and provide in-home care to Veterans who are often older, manage multiple medical conditions, and as a result have difficulty traveling to VA clinics for appointments (9, 10). HBPC programs are composed of interdisciplinary team members including, but not limited to nurses, physicians, social workers, and nurse practitioners (11). As HBPC Veterans often manage multiple serious illnesses, completion of life-sustaining treatment templates is a high priority to ensure Veterans' wishes for treatment are elicited and honored (2), and this is especially important in the last months of life (12). However, VA secondary data sources indicate that life-sustaining treatment template completion ranges from 0 to 100% across HBPC programs (13). The HBPC arm of the PERSIVED program aims to equip clinicians in HBPC programs with data and tools to document Veterans' life-sustaining treatment preferences to promote goal-concordant care. This is done through identifying facilitators and barriers to life-sustaining treatment template completion and applying evidence-based implementation strategies (14) to support teams in improving life-sustaining treatment template completion rates.

### PERSIVED program structure and HBPC program selection

The HBPC project of the PERSIVED program is composed of seven VA staff, including a physician, an implementation scientist, staff experienced in conducting implementation science, a social worker, and an advanced practice registered nurse who previously managed VA HBPC programs. The team was intentionally crafted to best support HBPC programs participating in PERSIVED. We selected HBPC programs to participate in PERSIVED from a pool of programs with lower than 50% life-sustaining treatment template completion rates for their HBPC Veteran caseload. An average HBPC team caseload is around 25–30 Veterans per nurse on an HBPC team, with three nurses on average per HBPC team (9). PERSIVED team members discussed eligible HBPC programs with the national HBPC program manager to identify appropriateness for participation, and HBPC facility program leadership were subsequently contacted and invited to participate. Thus far, HBPC programs who agreed to participate have identified at least two champions to coordinate efforts with the PERSIVED team. Champions can be any HBPC team member involved in conducting or documenting goals of care conversations. During the implementation period of PERSIVED, HBPC champions receive targeted implementation strategies (15) to improve life-sustaining treatment documentation rates.

## Implementation strategies

### Tailoring implementation strategies

In recent years, implementation scientists have highlighted the need for, and importance of, applying tailored implementation strategies to improve and inform implementation of programs and interventions in healthcare settings (16, 17). Challenges remain in translating the determinants scholars identify that impact implementation into the real world of clinical healthcare settings to address and improve upon programs and interventions. Kirchner et al. (14) ties in both the importance of conducting formative evaluations to uncover relevant determinants that highlight facilitators and barriers to a program's or intervention's success, and the need for tailoring the implementation strategy of facilitation to best support clinical teams and programs to improve upon processes (14). Other studies in recent years have examined facilitation strategies in implementation interventions in healthcare settings (18, 19). Further, the wide breadth and depth of implementation science literature in the past 15 years has shown the need to study and identify such determinants to improve upon implementation. Much of this scholarship has used frameworks like CFIR (consolidated framework for implementation research), REAIM (reach, effectiveness, adoption, implementation, maintenance framework), PARIHS (Promoting Action on Research Implementation in Health Services framework), PRISM (Practical, Robust Implementation and Sustainability. Model), and TICD, often to inform the tailoring of implementation strategies. However there remains a need to outline methodological approaches to applying implementation frameworks that use tailoring to inform facilitation.

### Implementation facilitation and audit with feedback in PERSIVED

In PERSIVED, two implementation strategies are applied: audit with feedback and implementation facilitation (14), which are commonly used in implementation of evidence-based practices in healthcare settings. To use audit with feedback, PERSIVED staff synthesize data on each HBPC program's life-sustaining treatment template completion rates and share these data with corresponding program champions, similar to our previous efforts assessing the implementation of the LSTDI in the VA (6, 20). This paper focuses on implementation facilitation in the PERSIVED program, which is a multi-faceted approach used for problem solving and providing support, and intervention context and characteristics of those delivering and receiving the intervention influence facilitation (21–23). Two PERSIVED team members act as external facilitators and two team members provide administrative support over video meetings with HBPC champions, thus partnering with HBPC programs to best support them in increasing documentation of life-sustaining treatment templates.

### Pre-implementation phase and application of the Tailored Implementation in Chronic Diseases framework using a rapid analytic approach

**Figure 1:**
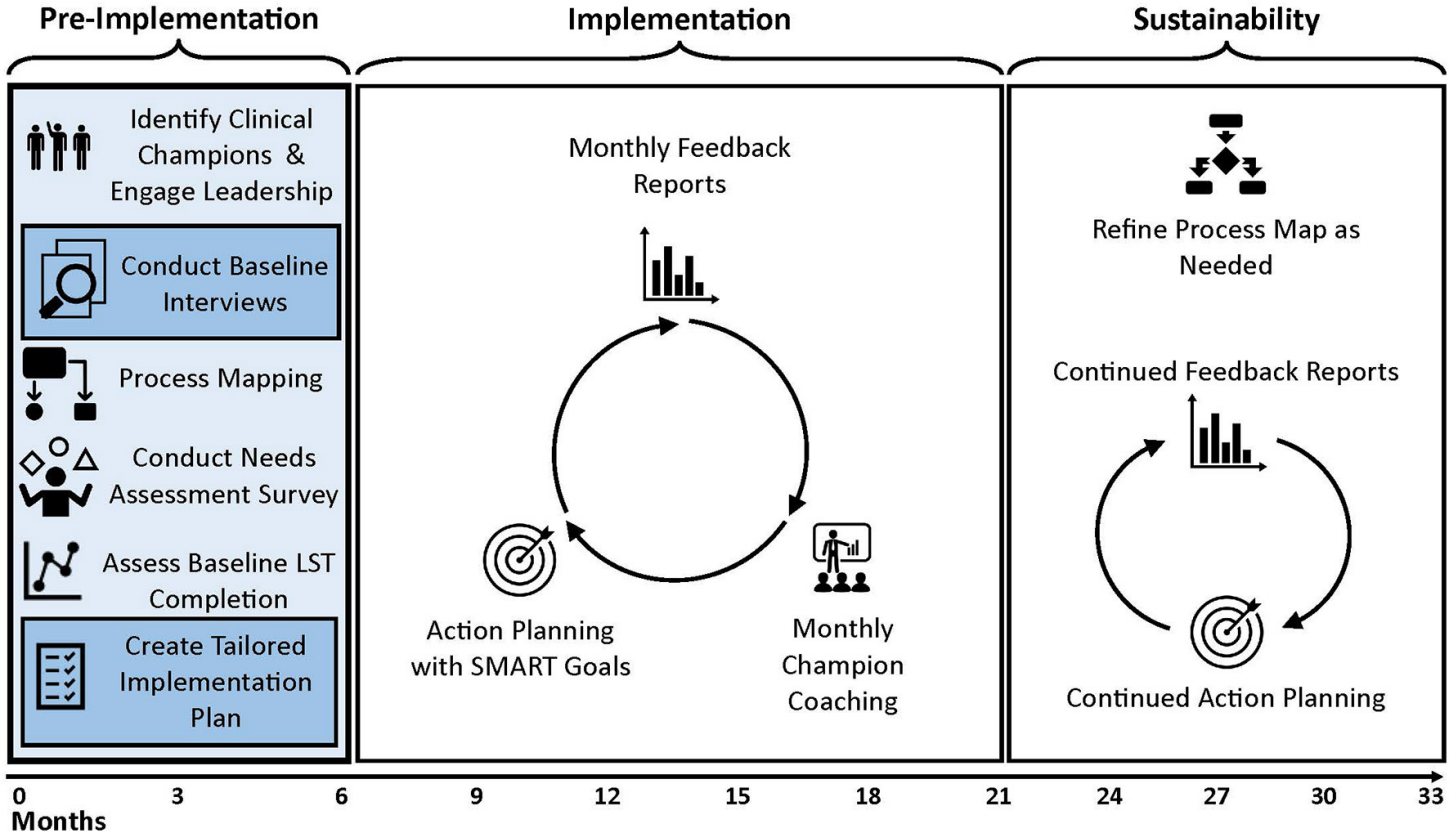
PERSIVED project timeline.

**Figure 2:**
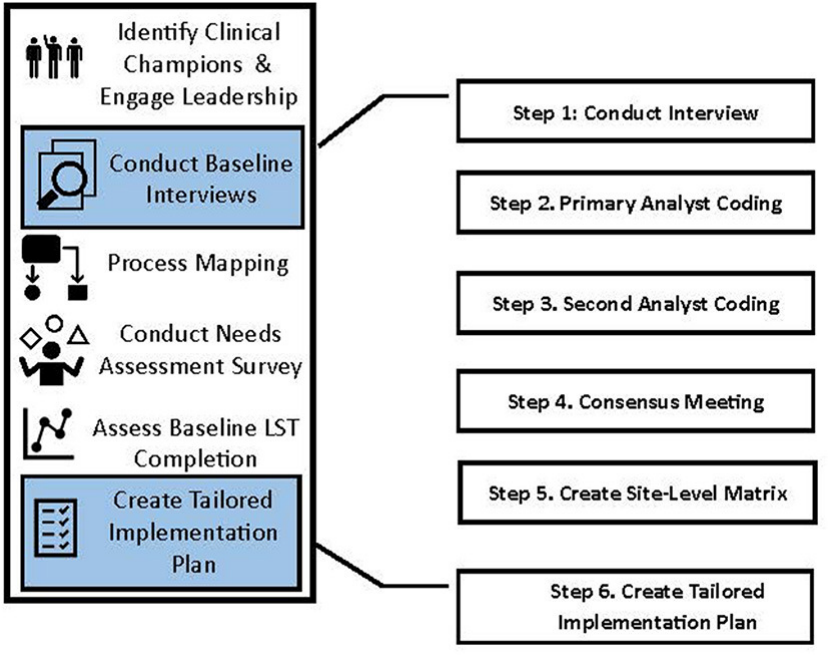
Pre-implementation.

Prior to beginning the PERSIVED implementation phase, each HBPC program participates in a five-month pre-implementation period. During the pre-implementation period, PERSIVED team members conduct semi-structured interviews, needs assessments, and process mapping with HBPC teams depicted in Figure 1. For purposes of this manuscript, we describe the methodological processes—rather than the results of the analyses—we use to conduct pre-implementation interviews, to analyze interview data, and to synthesize analyzed data to create tailored implementation plans for HBPC programs participating in PERSIVED (Figure 2). We conducted *N* = 37 semi-structured interviews with HBPC program champions and HBPC team members from seven different programs between September 2021 and March 2022. Interview participants included nurses, social workers, psychologists, primary care providers, dieticians, pharmacists, and program directors across seven HBPC programs.

The interviews gathered background information on current processes of conducting goals of care conversations and documenting life-sustaining treatment decisions, including assessing barriers, facilitators, baseline knowledge, skills, and resources (See Appendix 1 in Supplementary material for Interview Guide). PERSIVED staff then used findings from analyzing these interviews, guided by applying the TICD, to tailor PERSIVED intervention efforts to each HBPC program. To date, interviews range from 20 to 60 min and are recorded. Future manuscripts will describe findings from these interviews, as well as how we incorporated process maps and analysis of needs assessments.

The interview guide was designed and interview data were analyzed through application of the TICD framework (24). The TICD identifies 57 determinants that might influence practice or implementation of interventions like PERSIVED, and organizes determinants in seven domains: guideline factors, individual health professional factors, patient factors, professional interactions, incentives and resources, capacity for organizational change, and social, political, and legal factors (25). We selected the TICD because we felt it was the most appropriate framework to apply to study PERSIVED implementation as it combines aspects of both the CFIR and the Theoretical Domains Framework (TDF), in that it covers both organizational and individual determinants of implementation in a parsimonious manner (24). The PERSIVED implementation scientist (LH) adapted a rapid analytic approach based on previous implementation science approaches using the CFIR (26, 27), to create a team-based analytic approach to analyze interview data, which would then provide real-time results to PERSIVED facilitators to discuss with HBPC programs.

The rapid approach is beneficial because it allows PERSIVED team members to provide timely feedback and support to HBPC programs participating in the intervention, rather than using a traditional content analysis approach to analyzing interview data which is often more time consuming and laborious (26, 28). The implementation scientist trained four PERSIVED team members in this approach, all of whom had some level of experience applying the CFIR and other implementation science approaches. For training in applying the TICD to analyze interview data, five PERSIVED team members initially coded one interview that was conducted as a pilot interview with an HBPC team member from a non-intervention program. Analysts coded independently to the determinants in the TICD template, and then met over the course of three, one-hour meetings to reach consensus on what sections of interview data should be assigned to specific TICD determinants in the template. Following these rigorous discussions, the implementation scientist and analysts felt comfortable coding future interviews in pairs. This is the first project utilizing a rapid analysis approach to using the TICD.

## Steps to rapidly analyze qualitative data using the TICD

PERSIVED analysts created a template consisting of the 57 TICD determinants and definitions (Appendix 2 shows the Microsoft Word TICD template), and employed a team-based, rapid analytic approach to analyzing the data. This is similar to past studies which have also applied the TICD in pre-implementation phases of interventions (14, 29). Steps in applying the TICD to the PERSIVED baseline interview data to create tailored implementation facilitation plans are described below and outlined in Table 1.

**Table 1:** Steps of rapid analysis using the Tailored Implementation in Chronic Diseases framework.

Step 1. Conduct Interview Using Interview Guide in Appendix 1
• Two team members are present during the phone interviewer. One team member serves as the notetaker (primary analyst) and the other is the interviewer (secondary analyst) • Interviews are recorded • Notetaker/primary analyst takes detailed notes
Step 2. Primary Analyst Coding Using Template in Appendix 2
• 24-72 hours after the interview, the primary analyst codes the notes into the MS Word TICD template in Appendix 2 • Primary analyst flags sections of the notes to discuss during the consensus meeting with the secondary analyst (Figure 2) • Primary analyst listens to the recording if there are note sections that are unclear
Step 3. Secondary Analyst Coding Using Template in Appendix 2
• Interviewer acts as the secondary analyst • Secondary analyst edits and builds upon the primary analyst's template, going back to read notes and listen to recording as needed • Secondary analyst adds comments to discuss during consensus meeting • Secondary analyst writes summaries of each domain and organizes summaries as barriers, facilitators, and recommendation (Figure 2)
Step 4. Consensus Meeting Using Template in Appendix 2
• Primary and secondary analysts meet to review the TICD word template, settle any coding disagreements, and finalize the summaries drafted by the secondary analyst (Figure 2) • Primary analyst saves a final version of the TICD template with only the summaries
Step 5. Create Site-Level TICD Excel Matrix Using Template in Appendix 3
• Primary analyst creates a master matrix in excel that combines all the interview templates with final summaries for a specific HBPC site (Figure 2) • Primary analyst synthesizes summaries across the interviews and categorizes the summaries into facilitators, barriers, and recommendations • Qualitative team members review the site-level summaries during a consensus meeting
Step 6. Create Tailored Implementation Plan Using Template in Appendix 4
• Analysts use the facilitators, barriers, and recommendations from the master excel sheet to inform and tailor the site's implementation plan (Figure 2) • Analysts brainstorm implementation facilitation strategies to overcome barriers, build on facilitators, and incorporate recommendations

### Step 1: Conduct interview

All interviews are conducted over phone or video using Microsoft Teams, with two PERSIVED team members conducting the interview. One team member serves as the interviewer and secondary analyst and the other as the notetaker and primary analyst. Team members switch roles across interviews, with each team member serving as both interviewer and notetaker. All interviews are recorded and the notetaker/primary analyst takes detailed notes that are used for the TICD rapid analyses.

### Step 2: Primary analyst coding

Within 24–72 hours after the interview, the primary analyst codes the interview notes to the appropriate TICD determinants in the Microsoft Word TICD template (Appendix 2) (17, 25, 30). The primary analyst uses comments within Microsoft Word to flag sections to discuss during the consensus meeting with the secondary analyst. The primary analyst will listen to the interview recording if there are note sections that are unclear. Once finished, the primary analyst alerts the secondary analyst to complete their coding.

### Step 3: Secondary analyst coding

Once the primary analyst completes their coding, the secondary analyst edits and builds upon the primary analyst's template, going back to read notes and listen to the recording as needed. The secondary analyst adds comments to discuss during the consensus meeting. The secondary analyst then writes summaries of each domain and categorizes summaries as barriers, facilitators, or suggestions.

### Step 4: Consensus meeting

The primary and secondary coder meet to review the TICD template and settle any coding disagreements. The primary analyst reviews the summaries that were drafted by the secondary analyst and confirms their agreement or provides edits, if needed. The primary analyst then saves a final version of the TICD template with the agreed upon codes and summaries.

### Step 5: Create program-level TICD excel matrix

The primary analyst creates a master matrix in excel, depicted in Appendix 3, that combines all interview templates with final summaries for a specific HBPC program. The primary analyst synthesizes summaries across interviews into each specific TICD domain. The summaries are categorized as facilitators, barriers, and suggestions. The qualitative team then reviews the program-level summaries during a consensus meeting.

### Step 6: Create tailored implementation plan

The primary analyst then transfers the site-level summaries into an Implementation Plan (Appendix 4) that is divided by TICD domains. The summaries are categorized as barriers/challenges, facilitators/successes, and suggestions. An additional column (the resource/strategy to address barrier and leverage facilitator column) provides an area for the implementation team—including the two analysts and two external facilitators—to brainstorm and operationalize implementation facilitation activities that can address barriers and leverage facilitators, which leads to tailoring plans for implementation facilitation to the specific HBPC program. The focus on utilizing this column is based on a VA-produced implementation facilitation training manual that defines implementation facilitation activities (6, 31). The two external facilitators then use the summaries to guide their implementation facilitation activities, communicate appropriate points back to the programs, share resources, encourage goal setting, and troubleshoot problem solving related to improving rates of completion of life sustaining treatment templates.

## Discussion

The purpose of this manuscript is to outline how our team is applying the TICD framework (24) to inform program-specific implementation facilitation to improve completion of life-sustaining treatment templates for Veterans in HBPC programs participating in PERSIVED. Our approach was adapted from that of Nevedal et al.'s (26) and our team's experience applying the Consolidated Framework for Implementation Research to assess and evaluate large VA initiatives (7, 32).

This manuscript highlights our use of the TICD framework to analyze interview data gathered in the pre-implementation phase of PERSIVED, and the TICD is designed to be useful to inform development of interventions (33), as it is a combination of 12 different frameworks and checklists (24). In our work we have extended how the TICD has been previously used by having the TICD guide our analysis of baseline interview data with HBPC program participants, that in turn informs implementation facilitation with HBPC programs participating in the PERSIVED intervention. We have found this to be a useful and efficient analytic approach allowing for quick feedback to HBPC programs participating in PERSIVED.

We feel the major benefit to our analytic approach is using TICD rapidly, which to our knowledge is the first time this has been done. Using detailed notes taken by the primary analyst, rather than generating verbatim transcripts from recordings, saves money otherwise spent on transcription costs as well as time. Having the two analysts who conduct the interviews and take notes coding in pairs also saves time, and we feel this approach has proven both trustworthy and rigorous and matches well with our quality improvement process, where timely analysis has been necessary to inform the implementation-phase coaching calls with HBPC programs participating in PERSIVED. Therefore, we feel using the TICD has been an efficient approach to tailor implementation plans for HBPC programs participating in the PERSIVED program. As PERSIVED is a five-year program, the team will compare how implementation facilitation strategies are tailored across HBPC programs and program cohorts participating in PERSIVED, and the maintenance of those changes over time. This will allow us to identify best practices for improving life-sustaining treatment template completion rates and to share best practices with other VA HBPC programs.

## Limitations

While the rapid analytic method we are applying to analyzing interviews in the pre-implementation of PERSIVED is highly efficient and rigorous, it is not without limitations. One limitation is that two analysts are needed on each interview, so adequate staffing levels are important, and if the same pairs are able to work together consistently, consensus on assigning interview data to TICD determinants can be reached more efficiently. Additionally, planning for time to train analysts in applying the TICD is essential for them to be familiar with TICD determinants. This training must be focused and clear in order for analysts to successfully assign interview data to determinants and thus make useful meaning out of the data that can have valuable, practical use in implementation facilitation with participating PERSIVED HBPC teams.

## Conclusion

This manuscript describes the use of TICD framework to rapidly analyze pre-implementation interview data and create tailored implementation plans for HBPC programs participants in PERSIVED. We believe that this rapid approach can act as a practical guide for other implementation scientists, evaluators, and researchers to tailor to assessments, quality improvement projects, and evaluations of future interventions or initiatives.

## Data availability statement

The original contributions presented in the study are included in the article/Supplementary material, further inquiries can be directed to the corresponding author.

## Author contributions

LH, KM, and JK contributed to study concept and design, to the design of themethod, analysis, interpretation of data, and to drafting and revising the manuscript for important intellectual content. KM and JK contributed to acquisition of data. All authors confirm that neither the manuscript nor any parts of its content are currently under consideration or published in another journal, meet the criteria for authorship, and have approved the manuscript and agree with its submission to Frontiers in Health Services Research.

## Funding

This program was funded through the United States Department of Veterans Affairs Quality Enhancement Research Initiative (QUERI) program. VA QUERI funds are operational funds in the Veterans Health Administration. The Grant Number is QUE 20-015. Funds from this grant will cover the open access publications fees. The sponsor played no role in the design of this quality improvement project, project methods, recruitment, data collection, data analysis, or the preparation of this manuscript.

## Conflict of interest

The authors declare that the research was conducted in the absence of any commercial or financial relationships that could be construed as a potential conflict of interest.

## Publisher's note

All claims expressed in this article are solely those of the authors and do not necessarily represent those of their affiliated organizations, or those of the publisher, the editors and the reviewers. Any product that may be evaluated in this article, or claim that may be made by its manufacturer, is not guaranteed or endorsed by the publisher.

## Author disclaimer

The views expressed in this manuscript are those of the authors and do not necessarily reflect the views of the Department of Veterans Affairs or the United States government.

## Abbreviations

VA: Department of Veterans Affairs
PERSIVED: Preferences Elicited and Respected for Seriously Ill Veterans through Enhanced Decision-Making
HBPC: Home Based Primary Care
TICD: Tailored Implementation in Chronic Diseases
LSTDI: Life-Sustaining Treatment Decisions Initiative
QUERI: Department of Veterans Affairs Quality Enhancement Research Initiative
CFIR: Consolidated Framework for Implementation Research.

## References

[1] Foglia MB Lowery J Sharpe VA Tompkins P Fox E. A Comprehensive approach to eliciting, documenting, and honoring patient wishes for care near the end of life: the veterans health administration's life-sustaining treatment decisions initiative. Jt Comm J Qual Patient Saf. (2019) 45:47–56. 10.1016/j.jcjq.2018.04.007 30126715

[2] Sales AE Ersek M Intrator OK Levy C Carpenter JG Hogikyan R . Implementing goals of care conversations with veterans in VA long-term care setting: a mixed methods protocol. Implement Sci. (2016) 11:132. 10.1186/s13012-016-0497-0 27682236 PMC5041212

[3] Levy C Ersek M Scott W Carpenter JG Kononowech J Phibbs C . Life-sustaining treatment decisions initiative: early implementation results of a national veterans affairs program to honor veterans' care preferences. J Gen Intern Med. (2020) 35:1803–12. 10.1007/s11606-020-05697-2 32096084 PMC7280392

[4] Giannitrapani KF Walling AM Garcia A Foglia M Lowery JS Lo N . Pilot of the life-sustaining treatment decisions initiative among veterans with serious illness. Am J Hosp Palliat Med. (2021) 38:68–76. 10.1177/1049909120923595 32383388

[5] Documentation of Goals of Care Life-Sustaining Treatment Decisions through LST Progress Note. VA VSSC Data National Center for Ethics in Health Care. (2022). Available online at: https://reports.vssc.med.va.gov/ReportServer/Pages/ReportViewer.aspx?%2FPC%2FGoalsOfCare%2FGOCC_MAIN&rs%3ACommand=Render (accessed March 01, 2022).

[6] Carpenter JG Scott WJ Kononowech J Foglia MB Haverhals LM Hogikyan R . Evaluating implementation strategies to support documentation of veterans' care preferences. Health Serv Res. (2022) 5:734–43. 10.1111/1475-6773.13958 35261022 PMC9264454

[7] HaverhalsLM GilmanC ManheimC BauersC KononowechJ LevyC. Implementation of VA's life-sustaining treatment decisions initiative: facilitators and barriers to early implementation across seven VA medical centers. J Pain Symptom Manage. (2021) 62:125–133.e2. 10.1016/j.jpainsymman.2020.10.03433157178

[8] PERSIVED QUERI Program,. Veterans Health Administration. (2021). p. 1–2. Available online at: https://www.queri.research.va.gov/centers/PERSIVED.pdf (accessed March 31, 2022).

[9] VHA directive 1411. Home-Based Primary Care special population patient aligned care team program. (2017) p. 1–43.

[10] HaverhalsLM ManheimC GilmanC KaruzaJ OlsanT EdwardsST . Dedicated to the mission: strategies US department of veterans affairs home-based primary care teams apply to keep veterans at home. J Am Geriatr Soc. (2019) 67:2511–8. 10.1111/jgs.1617131593296

[11] GillespieSM ManheimC GilmanC KaruzaJ OlsanTH EdwardsST . Interdisciplinary team perspectives on mental health care in va home-based primary care: a qualitative study. Am J Geriatr Psychiatry. (2019) 27:128–37. 10.1016/j.jagp.2018.10.00630424995

[12] Batten A Cohen JH Foglia MB Alfandre D. Associations between the veteran health administration's life-sustaining treatment decisions initiative and quality of care at the end of life. J Palliat Med. (2022) 24:1057–63. 10.1089/jpm.2021.0488 35020477

[13] Wang H Berry B Wesgate S Intrator O. The Veterans Health Administration HBPC masterfile data dictionary. Geriatrics & Extended Care Data Analyses Center. Canandaigua, NY: Veterans Health Administration (2021).

[14] Kirchner JE Smith JL Powell BJ Waltz TJ Proctor EK. Getting a clinical innovation into practice: an introduction to implementation strategies. Psychiatry Res. (2020) 283:112467. 10.1016/j.psychres.2019.06.042 31488332 PMC7239693

[15] Lyon AR Coifman J Cook H McRee E Liu FF Ludwig K . The Cognitive Walkthrough for Implementation Strategies (CWIS): a pragmatic method for assessing implementation strategy usability. Implement Sci Commun. (2021) 2:1–16. 10.1186/s43058-021-00183-0 34274027 PMC8285864

[16] Wensing M Oxman A Baker R Godycki-Cwirko M Flottorp S Szecsenyi J . Tailored implementation for chronic diseases (TICD): a project protocol. Implement Sci. (2011) 6:103. 10.1186/1748-5908-6-103 21899753 PMC3179734

[17] Lescure D Haenen A de Greeff S Voss A Huis A Hulscher M. Exploring determinants of hand hygiene compliance in LTCFs: A qualitative study using Flottorps' integrated checklist of determinants of practice. Antimicrob Resist Infect Control. (2021) 10:1–11. 10.1186/s13756-021-00882-2 33446248 PMC7809817

[18] Moussa L Garcia-Cardenas V Benrimoj SI. Change facilitation strategies used in the implementation of innovations in healthcare practice: a systematic review. J Chang Manag. (2019) 19:283–301. 10.1080/14697017.2019.1602552

[19] Moussa L Benrimoj S Musial K Kocbek S Garcia-Cardenas V. Data-driven approach for tailoring facilitation strategies to overcome implementation barriers in community pharmacy. Implement Sci. (2021) 16:73. 10.1186/s13012-021-01138-8 34281587 PMC8290596

[20] Landis-Lewis Z Kononowech J Scott WJ Hogikyan R V Carpenter JG Periyakoil VS . Designing clinical practice feedback reports: three steps illustrated in Veterans Health Affairs long-term care facilities and programs. Implement Sci. (2020) 15:7. 10.1186/s13012-019-0950-y 31964414 PMC6975062

[21] Hartmann CW Engle RL Pimentel CB Mills WL Clark VA Keleher VC . Virtual external implementation facilitation: successful methods for remotely engaging groups in quality improvement. Implement Sci Commun. (2021) 2:66. 10.1186/s43058-021-00168-z 34158115 PMC8218278

[22] Perry CK Damschroder LJ Hemler JR Woodson TT Ono SS Cohen DJ. Specifying and comparing implementation strategies across seven large implementation interventions: a practical application of theory. Implement Sci. (2019) 14:1–13. 10.1186/s13012-019-0876-4 30898133 PMC6429753

[23] Powell BJ Waltz TJ Chinman MJ Damschroder LJ Smith JL Matthieu MM . A refined compilation of implementation strategies: results from the Expert Recommendations for Implementing Change (ERIC) project. Implement Sci. (2015) 10:21. 10.1186/s13012-015-0209-1 25889199 PMC4328074

[24] Flottorp SA Oxman AD Krause J Musila NR Wensing M Godycki-Cwirko M . A checklist for identifying determinants of practice: A systematic review and synthesis of frameworks and taxonomies of factors that prevent or enable improvements in healthcare professional practice. Implement Sci. (2013) 8:1–11. 10.1186/1748-5908-8-35 23522377 PMC3617095

[25] Zipfel N Horreh B Hulshof CTJ Suman A de Boer AGEM van der Burg-Vermeulen SJ. Determinants for the implementation of person-centered tools for workers with chronic health conditions: a mixed-method study using the Tailored Implementation for Chronic Diseases checklist. BMC Public Health. (2021) 21:1–19. 10.1186/s12889-021-11047-6 34098911 PMC8183322

[26] Nevedal AL Reardon CM Opra Widerquist MA Jackson GL Cutrona SL White BS . Rapid versus traditional qualitative analysis using the Consolidated Framework for Implementation Research (CFIR). Implement Sci. (2021) 16:1–12. 10.1186/s13012-021-01111-5 34215286 PMC8252308

[27] Damschroder LJ Reardon CM Opra Widerquist MA Lowery J. Conceptualizing outcomes for use with the Consolidated Framework for Implementation Research (CFIR): the CFIR Outcomes Addendum. Implement Sci. (2022) 17:7. 10.1186/s13012-021-01181-5 35065675 PMC8783408

[28] Gale RC Wu J Erhardt T Bounthavong M Reardon CM Damschroder LJ . Comparison of rapid vs in-depth qualitative analytic methods from a process evaluation of academic detailing in the Veterans Health Administration. Implement Sci. (2019) 14:1–12. 10.1186/s13012-019-0853-y 30709368 PMC6359833

[29] Hoffman AS Bateman DR Ganoe C Punjasthitkul S Das AK Hoffman DB . Development and field testing of a long-term care decision aid website for older adults: engaging patients and caregivers in user-centered design. Gerontologist. (2020) 60:935–46. 10.1093/geront/gnz141 31773140 PMC7456976

[30] Paudel R Ferrante S Woodford J Maitland C Stockall E Maatman T . Implementation of prostate cancer treatment decision aid in Michigan: a qualitative study. Implement Sci Commun. (2021) 2:1–10. 10.1186/s43058-021-00125-w 33676583 PMC7936475

[31] Ritchie M Dollar K Miller C Smith J Oliver K Kim B . Using Implementation Facilitation to Improve Healthcare (Version 3). (2020). Available online at: https://www.queri.research.va.gov/tools/Facilitation-Manual.pdf (Accessed January 15, 2021).

[32] Haverhals LM Sayre G Helfrich CD Battaglia C Aron D Stevenson LD . E-consult implementation: lessons learned using consolidated framework for implementation research. Am J Manag Care. (2015) 21:e640–7. Available online at: https://www.ncbi.nlm.nih.gov/pmc/articles/PMC4717483/26760426 PMC4717483

[33] Wensing M. The Tailored Implementation in Chronic Diseases (TICD) project: introduction and main findings. Implement Sci. (2017) 12:5. 10.1007/978-90-368-1732-5 28069029 PMC5223579

