# Supplementary material for: Applying the Tailored Implementation in Chronic Diseases framework to inform implementation of the Preferences Elicited and Respected for Seriously Ill Veterans through enhanced decision-making program in the United States Veterans Health Administration
Source: Front Health Serv. 2022 Sep 2;2:935341. doi: 10.3389/frhs.2022.935341 (PMC10012641; doi:10.3389/frhs.2022.935341)
Supplement: Supplementary file 1 [file Data_Sheet_1.docx]

**Interviewee Name:**

**VA Team Role:**

**Site Name and Program (HBPC or CNH):**

**Interviewer Name:**

**Note Taker Name:**

**Date of interview:**

**Introduction to the Interview and Permission to Record**

Thank you for participating in the interview today! You might remember that we sent you a brief summary of the PERSIVED program and the Life-Sustaining Treatment Decisions Initiate (LSTDI) prior to this interview. Do you have any questions about the summary? Any points you would like me to clarify? [IF INTERVIEWEE DID NOT READ SUMMARY BEFOREHAND, SHARE DOCUMENT ONLINE IN TEAMS AND REVIEW BRIEFLY].

The purpose of this interview is to learn about your perceptions of how the LSTDI has been implemented in your HBPC/CNH program and to help us understand how the PERSIVED program can best support programs to increase the number of goals of care conversations and completed LST templates for your Veterans, improve the quality of the goals of care conversation, and ensure that Veterans’ preferences for LST are honored.

The interview will last about 30-60 minutes. With your permission, I would like to audio-record our conversation; this helps me to listen better and ensures an accurate and complete record. Everything you tell us will be confidential. To protect your privacy, we won't connect your name with anything that you say.

At any time during our conversation, feel free to let me know if you have any questions or if you would rather not answer any specific question. You can also stop the interview at any time for any reason. Please remember that we want to know what you think and believe; there are no right or wrong answers.

Is it OK if I audiotape this interview today? [if yes] Thank you. I will now turn on the recorder and ask you to restate your agreement. **[Turn on recording equipment.]** The recorder is now switched on. Do you agree to the recording of this interview? Thanks again.

Do you have any questions before we begin?

**Background**

I'd like to begin by asking you some questions about your current job.

1. What is your position at [SITE]?
   1. What are your major responsibilities in your current position?
2. How long have you worked at [SITE]?
   1. How long have you worked in your current position?
3. Have you worked in other programs or at other VAs?
   1. What were those roles?
   2. How long did you hold these other positions?
   3. How long have you worked at the VA overall?

**Familiarity, experience, and training with LSTDI**

1. As I mentioned, our program focuses on the national VA Life Sustaining Treatment Decisions Initiative (LST Initiative). What do you know about this initiative? How familiar were you with the initiative before I described it?
2. What is your role in the LST initiative on your team or facility? (probes: *Are you involved with conducting goals of care conversations, completing the LST template and order set, teaching other about LST, quality improvement activities around LST?)*
3. Are you familiar with the LST note and orders template?
4. Do you actually complete either the note or orders template?
5. ***If yes,*** What has worked well?
6. What has been challenging?
7. What would you like to change if you could?

7. Have you participated in any national or local LSTDI trainings?

If ‘yes”: which type(s) of training did you attend? (Online/face to face, full day/half day, national training, visit level training, or facility level training etc.)

- 1. Can you provide an estimate of how many of your [HBPC or CNH] team members have attended a training? [If “yes”, ask interviewee to provide a percentage]
  2. How effective do you think these trainings were in increasing you or the team’s confidence and skills in conducting goals of care conversations and completing the LST templates? [Effectiveness, Implementation]

1. Many local, VISN-level and national groups have developed resources to support clinicians who are implementing the LST initiative. Many of these come from the National Center for Ethics in Health Care. Are you familiar with any of these resources?

**If “yes”,** which ones did you use and how helpful were they in implementing and sustaining the LST I initiative? (Below are PROMPTS)

- 1. Monthly support calls/LST “office hours
  2. VHA Handbook 1004.03 (the LSTDI Handbook)
  3. LSTDI educational sheets and sharing them with Veterans and their families
  4. Online educational modules produced by the NCEHC
  5. Feedback reports from the LTC QUERI
  6. Podcasts available on the VA LST DI webpage
  7. Skills training resources for staff and providers
  8. Other_______________________________________________

**Team processes and challenges to completing LST template**

Now, I want to ask you a bit more about how you and your team are doing with completing goals of care conversations and the LST template.

1. Tell us about your and/or your team’s experience conducting goals of care conversations in HBPC/CNH.
   1. Please describe your HBPC/CNH team process for conducting goals of care conversations with veterans were seriously ill.
   2. How do you feel about your team’s ability to conduct goals of care conversations with your Veterans?
      1. How do you feel about your team’s ability to have goals of care conversations with Veterans’ surrogate decision-makers?
   3. Thinking about the cultural / ethnic makeup of the population your team serves; do you find your experience with discussing or conducting goals of care conversations differs between any of these groups?
      1. How confident are you or your team members in discussing GOC with veterans from diverse groups?
   4. Other challenges? With Veterans/families or providers
   5. Other strategies used?
2. How is the LST template completion going in your program?
   1. How are providers doing with LST template completion?
   2. How do you feel about your team’s ability to complete the LST template?
   3. *Possible prompts:* How well do you think your team communicates with patients about LST/completes LST?
   4. How comfortable do you think your team is with communicating about/completing LST templates?
   5. What other factors have influenced LST template completion?
   6. For example, any factors related to Veterans/families or providers? Cerner transition?
3. Have you or your [HBPC/CNH] team members established any informal or formal processes to ensure that veterans have an opportunity to engage in goals of care discussions and complete the LST template? ***If yes***, can you describe those processes to me? [e.g., embedded in an admission visit, follow-up visit, designated staff to conduct the conversations and/or complete the LST template]
   1. Does your program have a process for reviewing or updating LST preferences and templates?

**Local and Team support for LSTDI**

Now I want to ask you about the resources and support that you have received, or may still need, to successfully implement the LSTDI.

1. In comparison to other VA initiatives that are going on right now, how has LST completion been prioritized for your HBPC/CNH team?
   1. How has leadership been involved in these efforts?
   2. How do you think leadership support has influenced LST template completion, if at all?
   3. Do you feel like you have the support overall you need to conduct goals of care conversations and complete the LST templates? Why or why not?
2. Do you consider that the LST initiative a high priority on your team? Why or why not?
3. How has the COVID-19 pandemic affected conducting goals of care conversations and completing the LST templates?
4. Anything else that you think we should be aware of about conducting goals of care conversations, completing the LST templates?

**Translation of treatment preferences into SAPOs**

1. Before we begin the next set of questions, we wanted to ask if you’re familiar with SAPOs or state authorized portable orders?

If no- read this section:

- SAPOs are also referred to as Physicians Orders for Life-Sustaining Treatment [POLST], Medical Orders for Scope of Treatment [MOST] or a similar title, depending on the state. In HBPC, you may refer to them as “refrigerator orders” because patients who are cared for in their homes are instructed to post them on their refrigerators for easy access by EMTs and other healthcare providers. SAPOs are particularly important for Veterans in the Community Nursing Home (CNH) and many Veterans in Home-Based Primary Care (HBPC) programs who receive some of their care in community hospitals. The NCEHC encourages VA providers to translate VA LST orders into *SAPOs* so the Veterans’ preferences can be documented and honored in non-VA settings. (There is a SAPO-related item on the new LST template that will be rolled out in the coming months.)

1. Do you think that SAPOs are regularly completed for veterans in your [HBPC/CNH] program who may receive care outside of the VA?

***If yes***, What are the factors that facilitate their completion? [A particular champion on the team? Expectations/pressure from community nursing homes or other community agencies? State requirements? Facility level directives or expectations? Other?]

***If no***, what are the factors that impede completion of a SAPO? [Resistance from team members? Lack of knowledge among team members about completing a SAPO? Expectation/evidence/belief that SAPOs are or should be completed by the community provider? Other?]

1. Have you or your [HBPC/CNH] team members established any informal or formal processes to translate Veterans’ LST preferences into a SAPO? ***If yes***, can you describe those processes to me? [e.g., embedded in an admission visit, follow-up visit, designated staff to conduct the conversations and/or complete the SAPO?]
2. How do you communicate these preferences to community providers (e.g., CNH program - community nursing homes, CCNs; HBPC – community hospitals)?
3. Can you describe any regulatory or legal factors that promote or hinder the completion of either LST templates or SAPOs and communicating them to community providers?
4. Are there other people on your team or in your facility we should talk with so we can understand the factors that promote or impede completion of goals of care conversations, LST templates, or SAPOs? ***If yes***, ask the interviewee for names, titles, and contact information.
