# Supplementary material for: Applying the Tailored Implementation in Chronic Diseases framework to inform implementation of the Preferences Elicited and Respected for Seriously Ill Veterans through enhanced decision-making program in the United States Veterans Health Administration
Source: Front Health Serv. 2022 Sep 2;2:935341. doi: 10.3389/frhs.2022.935341 (PMC10012641; doi:10.3389/frhs.2022.935341)
Supplement: Supplementary file 2 [file Data_Sheet_2.docx]

| **Domain/ Construct** | **Definition** | **Quotes** |
| --- | --- | --- |
| **1.Guideline/EBP/ Innovation factors** | | |
| **1a. Recommendation [**Guidelines Factors**]** | | |
| - Quality of evidence | How confident we are in the estimates of effects |  |
| - Strength of recommendation | How confident we are that the desirable effects of adherence to the recommendation outweigh the undesirable effects |  |
| - Clarity | The clearness of the target population, the settings in which the recommendation is to be used and the recommended action |  |
| - Cultural appropriateness | The extent to which the recommendation is suitable in the social context where it is being implemented |  |
| - Accessibility of the recommendation | How accessible the guideline or recommendation is |  |
| - Source of the recommendation | The organization(s) and people that made the recommendation |  |
| - Consistency with other guidelines | The extent to which the recommendation is consistent with recommendations in other guidelines with which the targeted healthcare professionals might be familiar |  |
| **1b. Recommended clinical intervention [**Guideline Factors**]** | | |
| - Feasibility | The extent to which the recommended clinical intervention is practical |  |
| - Accessibility of the intervention | The extent to which the recommended clinical intervention is accessible |  |
| **1c. Recommended behavior [**Guideline Factors**]** | | |
| - Compatibility | The extent to which the recommended behavior fits with current practices |  |
| - Effort | The amount of effort required to change or adhere |  |
| - Trialability | The ability to try out the recommended behavior |  |
| - Observability | The degree to which benefits of the recommended behavior are visible |  |
| **2. Individual health professional factors** | | |
| **2a. Knowledge and skills [**Individual health professional factors**]** | | |
| - Domain knowledge | The extent to which the targeted healthcare professionals have pre-existing knowledge or expertise about the targeted condition |  |
| - Awareness and familiarity with the recommendation | The extent to which the targeted healthcare professionals are aware of and familiar with the recommendation |  |
| - Knowledge about own practice | The extent to which the targeted healthcare professionals are aware of their own practice in relationship to the recommended practice |  |
| - Skills needed to adhere | The extent to which the targeted health professionals have skills that they need to adhere |  |
| **2b. Cognitions (including attitudes)** [Individual health professional factors] | |  |
| - Agreement with recommendations | The extent to which the targeted healthcare professionals agree with the recommendation |  |
| - Attitudes towards guidelines in general | The perceptions that the targeted healthcare professionals have regarding guidelines in general |  |
| - Expected outcome | The extent to which the targeted healthcare professionals believe that adherence with the recommendation will lead to desired outcomes |  |
| - Intention and motivation | The extent to which the targeted healthcare professionals intend to adhere and are motivated to do so |  |
| - Self-efficacy | The targeted healthcare professionals’ self-perceived competence or confidence in their abilities |  |
| - Learning style | The preferred ways in which the targeted healthcare professionals learn |  |
| - Emotions | The extent to which emotions affect adherence |  |
| **2c. Professional behavior** [Individual health professional factors] | | |
| - Nature of the behavior | Characteristics of the behavior, including: frequency of performance for a patient, frequency of performance for a population of patients, the degree of habit or automaticity, whether it is within a sequence of other behaviors that have to be performed, and whether it is performed by one person or by different people |  |
| - Capacity to plan change | The extent to which the targeted healthcare professionals have the capacity to plan necessary changes in order to adhere |  |
| - Self-monitoring or feedback | The extent to which the targeted healthcare professionals have the capacity for self-monitoring or feedback to reinforce adherence with the recommendation |  |
| **Patient Factors** | | |
| - Patient needs | Real or perceived needs and demands of the patient |  |
| - Patient beliefs and knowledge | Patients’ beliefs or knowledge or ability to learn, or the targeted healthcare professionals ‘ability or perceived ability to inform or teach patients necessary knowledge and skills |  |
| - Patient preferences | Patients’ values in relationship to professional values or those in the recommendation |  |
| - Patient motivation | The targeted healthcare professionals’ ability or perceived ability to motivate patients to adhere |  |
| - Patient behavior | Patient behaviours that motivate or demotivate adherence with the recommendation |  |
| **Professional interactions** | | |
| - Communication and influence | The extent to which the targeted healthcare professionals’ adherence is influenced by professional opinions and communication |  |
| - Team processes | The extent to which professional teams or groups have the skills needed to adhere and interact in ways that facilitate or hinder adherence |  |
| - Referral processes | Processes for transferring patients and communication between different levels of care, between health and social services, and between the targeted healthcare professionals and targeted patients |  |
| **Incentives and resources** | | |
| - Availability of necessary resources | The extent to which the resources that are needed to adhere are available |  |
| - Financial incentives and disincentives | The extent to which patients, individual health professionals and organizations have financial incentives or disincentives to adhere |  |
| - Nonfinancial incentives and disincentives | The extent to which patients, individual health professionals and organizations have nonfinancial incentives or disincentives to adhere |  |
| - Information system | The extent to which the information system facilitates or hinders adherence |  |
| - Quality assurance and patient safety systems | The extent to which existing quality assurance or patient safety systems facilitate or hinder adherence |  |
| - Continuing education system | The extent to which the continuing education system facilitates or hinders adherence |  |
| - Assistance for clinicians | The extent to which clinicians have the assistance they need to adhere |  |
| **Capacity for organizational change** | | |
| - Mandate, authority, accountability | The mandate, authority and accountability for making necessary changes |  |
| - Capable leadership | The extent to which clinical leaders or managers are capable of making necessary changes |  |
| - Relative strength of supporters and opponents | The extent of support and opposition to necessary changes |  |
| - Regulations, rules, policies | The extent to which organizational regulations, rules or policies facilitate or hinder necessary changes |  |
| - Priority of necessary change | The relative priority given to making necessary changes |  |
| - Monitoring and feedback | The extent to which monitoring and feedback are needed at organizational level and available to sustain necessary changes (including evaluations of improvement programs) |  |
| - Assistance for organizational changes | The extent to which external support is needed and available for necessary changes |  |
| **Social, political and legal factors** | | |
| - Economic constraints on the healthcare budget | Limits on the total healthcare budget or its growth |  |
| - Contracts | The extent to which contracts may affect implementation of necessary changes |  |
| - Legislation | The extent to which legislation may affect implementation of necessary changes |  |
| - Payer or funder policies | The extent to which payer or funder policies may affect implementation of necessary changes |  |
| - Malpractice liability | The extent to which malpractice liability may affect implementation of necessary changes |  |
| - Influential people | The extent to which influential people may affect implementation of necessary changes |  |
| - Corruption | The extent to which corruption may affect implementation of necessary changes |  |
| - Political stability | The extent to which political stability may affect implementation of necessary changes |  |
| **Inductive Constructs** | | |
| **Suggestions for Successful Uptake/ Implementation** | A suggestion provided during baseline interview on how PERSIVED can best support uptake of LSTDI during implementation. |  |
