# Supplementary material for: Applying the Tailored Implementation in Chronic Diseases framework to inform implementation of the Preferences Elicited and Respected for Seriously Ill Veterans through enhanced decision-making program in the United States Veterans Health Administration
Source: Front Health Serv. 2022 Sep 2;2:935341. doi: 10.3389/frhs.2022.935341 (PMC10012641; doi:10.3389/frhs.2022.935341)
Supplement: Supplementary file 4 [file Data_Sheet_4.docx]

| **Domain/ Construct** | **Barriers/ Challenges** | **Facilitators / Successes** | **Resource/ Strategy to Address Barrier and Leverage Facilitator** |
| --- | --- | --- | --- |
| **1.Guideline/EBP/ Innovation factors** | | |  |
| **1a. Recommendation [**Guidelines Factors**]** | | |  |
| - Quality of evidence |  |  |  |
| - Strength of recommendation |  |  |  |
| - Clarity |  |  |  |
| - Cultural appropriateness |  |  |  |
| - Accessibility of the recommendation |  |  |  |
| - Source of the recommendation |  |  |  |
| - Consistency with other guidelines |  |  |  |
| **1b. Recommended clinical intervention [**Guideline Factors**]** | | |  |
| - Feasibility |  |  |  |
| - Accessibility of the intervention |  |  |  |
| **1c. Recommended behavior [**Guideline Factors**]** | | |  |
| - Compatibility |  |  |  |
| - Effort |  |  |  |
| - Trialability |  |  |  |
| - Observability |  |  |  |
| **2. Individual health professional factors** | | |  |
| **2a. Knowledge and skills [**Individual health professional factors**]** | | |  |
| - Domain knowledge |  |  |  |
| - Awareness + familiarity with recommendation |  |  |  |
| - Knowledge about own practice |  |  |  |
| - Skills needed to adhere |  |  |  |
| **2b. Cognitions (including attitudes)** [Individual health professional factors] | |  |  |
| - Agreement with recommendations |  |  |  |
| - Attitudes towards guidelines in general |  |  |  |
| - Expected outcome |  |  |  |
| - Intention and motivation |  |  |  |
| - Self-efficacy |  |  |  |
| - Learning style |  |  |  |
| - Emotions |  |  |  |
| **2c. Professional behavior** [Individual health professional factors] | | |  |
| - Nature of the behavior |  |  |  |
| - Capacity to plan change |  |  |  |
| - Self-monitoring or feedback |  |  |  |
| **Patient Factors** | | |  |
| - Patient needs |  |  |  |
| - Patient beliefs + knowledge |  |  |  |
| - Patient preferences |  |  |  |
| - Patient motivation |  |  |  |
| - Patient behavior |  |  |  |
| **Professional interactions** | | |  |
| - Communication + influence |  |  |  |
| - Team processes |  |  |  |
| - Referral processes |  |  |  |
| **Incentives and resources** | | |  |
| - Availability of necessary resources |  |  |  |
| - Financial incentives and disincentives |  |  |  |
| - Nonfinancial incentives and disincentives |  |  |  |
| - Information system |  |  |  |
| - Quality assurance + patient safety systems |  |  |  |
| - Continuing education system |  |  |  |
| - Assistance for clinicians |  |  |  |
| **Capacity for organizational change** | | |  |
| - Mandate, authority, accountability |  |  |  |
| - Capable leadership |  |  |  |
| - Relative strength of supporters + opponents |  |  |  |
| - Regulations, rules, policies |  |  |  |
| - Priority of necessary change |  |  |  |
| - Monitoring and feedback |  |  |  |
| - Assistance for organizational changes |  |  |  |
| **Social, political, and legal factors** | | |  |
| - Economic constraints on the healthcare budget |  |  |  |
| - Contracts |  |  |  |
| - Legislation |  |  |  |
| - Payer or funder policies |  |  |  |
| - Malpractice liability |  |  |  |
| - Influential people |  |  |  |
| - Corruption |  |  |  |
| - Political stability |  |  |  |
